# The interaction between autophagy and the epithelial-mesenchymal transition mediated by NICD/ULK1 is involved in the formation of diabetic cataracts

**DOI:** 10.1186/s10020-022-00540-2

**Published:** 2022-09-14

**Authors:** Jiyuan Ma, Wei Ye, Yunshu Yang, Tong Wu, Yafen Wang, Ji Li, Rui Pei, Mengmei He, Luning Zhang, Jian Zhou

**Affiliations:** 1 Department of Ophthalmology, Eye Institute of PLA, Xijing Hospital, Fourth Military Medical University, 127 West Changle Road, Xi’an, 710032 Shaanxi People’s Republic of China; 2 Department of Burns and Cutaneous Surgery, Xijing Hospital, Fourth Military Medical University, Xi’an, Shaanxi People’s Republic of China; 3 Department of Transfusion Medicine, Xijing Hospital, Fourth Military Medical University, Xi’an, Shaanxi People’s Republic of China

**Keywords:** Diabetic cataract, Lens epithelial cells, Autophagy, Epithelial-mesenchymal transition, Notch signaling pathway, AKT/mTOR/ULK1 signaling pathway

## Abstract

**Background:**

Cataracts are the leading cause of blindness and a common ocular complication of diabetes. The epithelial-mesenchymal transition (EMT) of lens epithelial cells (LECs) and altered autophagic activity occur during the development of diabetic cataracts. The disturbed interaction of autophagy with EMT in LECs stimulated by high glucose levels may participate in cataract formation.

**Methods:**

A rat diabetic cataract model induced by streptozotocin (STZ) and human lens epithelial cells (HLE-B3) stimulated with a high glucose concentration were employed in the study. These models were treated with rapamycin (an inhibitor of mammalian target of rapamycin (mTOR)), and N-(N-[3,5-difluorophenacetyl]-1-alanyl)-S-phenylglycine t-butyl ester (DAPT, an inhibitor of γ-secretase) alone or in combination. Lens opacity was observed and photographed under a slit-lamp microscope. Histological changes in paraffin sections of lenses were detected under a light microscope after hematoxylin and eosin staining. Alterations of autophagosomes in LECs were counted and evaluated under a transmission electron microscope. The expression levels of proteins involved in the EMT, autophagy, and the signaling pathways in LECs were measured using Western blotting and immunofluorescence staining. Cell migration was determined by performing transwell and scratch wound assays. Coimmunoprecipitation (Co-IP) was performed to verify protein-protein interactions. Proteins were overexpressed in transfected cells to confirm their roles in the signaling pathways of interest.

**Results:**

In LECs, a high glucose concentration induces the EMT by activating Jagged1/Notch1/Notch intracellular domain (NICD)/Snail signaling and inhibits autophagy through the AKT/mTOR/unc 51-like kinase 1 (ULK1) signaling pathway in vivo and in vitro, resulting in diabetic cataracts. Enhanced autophagic activity induced by rapamycin suppressed the EMT by inducing Notch1 degradation by SQSTM1/p62 and microtubule-associated protein light chain 3 (LC3) in LECs, while inhibition of the Notch signaling pathway with DAPT not only prevented the EMT but also activated autophagy by decreasing the levels of NICD, which bound to ULK1, phosphorylated it, and then inhibited the initiation of autophagy.

**Conclusions:**

We describe a new interaction of autophagy and the EMT involving NICD/ULK1 signaling, which mediates crosstalk between these two important events in the formation of diabetic cataracts. Activating autophagy and suppressing the EMT mutually promote each other, revealing a potential target and strategy for the prevention of diabetic cataracts.

## Background

Approximately 500 million people worldwide suffer from diabetes, and this number is expected to increase by 25% in 2030 and 51% in 2045 (Saeedi et al. 2019). As a common ocular complication, diabetic cataracts are one of the main causes of vision impairment in patients with diabetes (Harding et al. 1993). The heavy socioeconomic burden (Lee and Afshari 2017) caused by cataract surgery and the intraoperative and postoperative complications of diabetic cataracts (Chan et al. 2010) should not be ignored. Therefore, the elucidation of the pathogenesis of diabetic cataracts to prevent or delay cataract formation using various treatments is necessary.

The establishment, maintenance and function of lens epithelial cells (LECs) are responsible for lens transparency (Martinez and Iongh 2010). The epithelial-mesenchymal transition (EMT) involves a loss of the epithelial characteristics of epithelial cells and acquisition of mesenchymal characteristics upon exposure to various external stimuli (Dongre and Weinberg 2019). Various studies have showed that LECs undergo the EMT under high glucose conditions in vitro or in cataracts of individuals with diabetes mellitus (Zhang et al. 2017; Li et al. 2019; Han et al. 2018; Du et al. 2017). EMT activation leads to the disruption of cell-cell junctions and cell polarity, degradation of the basement membrane, and extracellular matrix reorganization, as manifested by decreased expression of epithelial markers such as E-cadherin and ZO-1 and increased expression of mesenchymal markers such as α-SMA, N-cadherin and vimentin (Dongre and Weinberg 2019).

The Notch signaling pathway is known to induce the EMT in both normal and neoplastically transformed tissues (Dongre and Weinberg 2019). After the interactions between membrane-bound ligands (known as Jagged in mammals) and Notch receptors (known as Notch1, 2, 3, 4 in mammals), the Notch signaling pathway is activated, and Notch receptors are cleaved by γ-secretase, releasing the Notch intracellular domain (NICD), which translocates to the nucleus and promotes transcription (Bray 2006). Emerging evidence has indicated that the Notch signaling pathway regulates the EMT of LECs induced by TGF-β2 (Chen et al. 2017; Han et al. 2019), but researchers have not clearly determined whether the Notch signaling pathway participates in the high glucose-stimulated EMT of LECs.

Autophagy is a conserved process by which some portions of cells are degraded in the lysosome to sustain cellular homeostasis (Mizushima and Levine 2020). The typical process of autophagy includes four stages: initiation, elongation, maturation, and degradation (Chen et al. 2012). Microtubule-associated protein light chain 3 (LC3) is a key protein contributing to the major steps of autophagy (Huang and Liu 2015). In mammals, the conversion of LC3 from a freely diffuse form, LC3I, to a membrane-anchored, lipidated form, LC3II, is a characteristic signature of autophagic membranes (Dikic and Elazar 2018). SQSTM1/p62, a selective autophagic receptor, binds to both ubiquitylated proteins via its ubiquitin-associated (UBA) domain and LC3 via its LC3-interacting region (LIR) and directs cargo to autophagosomes for degradation (Rogov et al. 2014). The expression of this molecule is negatively correlated with autophagic activity (Johansen and Lamark 2011). Mammalian target of rapamycin (mTOR), a serine/threonine protein kinase, is an important regulator of stress-induced autophagy. Macroautophagy is negatively regulated by the PI3K/AKT/mTOR signaling pathway (Li et al. 2016). mTOR phosphorylates both unc-51-like kinase 1 (ULK1) and ATG13. ULK1 is inactivated after phosphorylation at Ser757; thus, autophagy is initiated (Kim et al. 2011). Deficiencies in autophagy may lead to various diseases, including neurodegeneration, inflammatory disorders, and cancer (Levine and Kroemer 2019).

Autophagy also plays a core role in lens development and the maintenance of lens transparency. The formation of an organelle-free zone (OFZ) is required for lens transparency. Studies have showed that the inactivation of MAPK/JNK signaling mediates the upregulation of autophagy proteins in the lens through the inhibition of mTOR-RPTOR signaling, leading to the autophagic loss of organelles and the subsequently formation of an OFZ (Basu et al. 2014). The presence of autophagic vesicles containing mitochondria in LECs, immature lens fiber cells and during the early stages of lens fiber cell differentiation has been documented, indicating that autophagy occurs throughout the development of the lens (Costello et al. 2013). Numerous studies have reported that dysregulation of autophagy is involved in the formation of different types of cataracts, such as congenital cataracts (Wignes et al. 2013; Chen et al. 2011), age-related cataracts (Morishita et al. 2013; Zhou et al. 2016), and diabetic cataracts (Liu et al. 2020; Chen et al. 2021). We speculate that autophagy is activated in normal LECs and that the loss of autophagy may result in alterations in lens development, loss of lens homeostasis and cataract formation.

Complex crosstalk between autophagy and EMT signaling pathways has been identified. EMT-related signaling pathways may trigger or suppress autophagy. Kiyono et al. (Kiyono et al. 2009) have suggested that TGF-β2 initiates autophagy via Smad and non-Smad pathways to promote human hepatocellular carcinoma cell invasion. However, Nopparat et al. (Nopparat et al. 2017) have showed that NF-κB, a key regulator of the EMT, suppresses autophagic activity by inhibiting Beclin-1, an initiator of autophagy. Autophagy-regulated signaling pathways may induce or inhibit the EMT process. According to Li et al. (Li et al. 2013), the induction of autophagy promotes the EMT in hepatocellular carcinoma cells in a TGF-β/Smad3 signaling-dependent manner. Nevertheless, Wang et al. (Wang et al. 2019) (Han et al. 2019) have reported that the inhibition of autophagy induces the EMT in RAS-mutated cells by activating the NF-κB pathway via SQSTM1/p62. As showed in our previous study, inhibition of autophagy enhanced the EMT in HLE-B3 cells under high glucose conditions via interactions of SQSTM1/p62 and Snail (Li et al. 2020). Nonetheless, other possible mechanisms regulating autophagy and EMT remain to be discovered.

In this study, we employed a diabetic rat model induced by streptozotocin (STZ) and human lens epithelial HLE-B3 cells stimulated with high glucose concentrations to observe the interaction of autophagy and the EMT. Mechanistically, the EMT is induced in LECs by high glucose concentrations via the Notch signaling pathway in vivo and in vitro, and autophagy is inhibited via the AKT/mTOR/ULK1 signaling pathway. Decreased autophagic degradation of Notch1 through SQSTM1/p62 and LC3 leads to the accumulation of NICD in the cytoplasm. Then, NICD binds to ULK1 and phosphorylates it, inhibiting of autophagy.

## Materials and methods

### Diabetic cataract model and treatment

SPF-grade male Sprague-Dawley rats (6–8 weeks, 180–240 g) were purchased from the Laboratory Animal Center of the Fourth Military Medical University. Rats were housed on 12-h/12-h light/dark cycles at a temperature of 25 ± 2 °C and a humidity of 59–61%. All animal procedures performed in this study were approved by the Animal Care Committee of the Fourth Military Medical University according to the guidelines in the Association for Research in Vision and Ophthalmology Statement. Sixty rats were included in this study.

Sixty rats (n = 60) were randomly divided into a normal control (NC) group (n = 15) and a diabetes mellitus group (n = 45). Diabetes mellitus was induced by administering an intraperitoneal injection of 1% STZ (60 mg/kg, Sigma-Aldrich, St. Louis, MO, USA) dissolved in citric acid-sodium citrate buffer (pH = 4.2–4.5). Rats in the NC group were injected with the corresponding volume of citric acid-sodium citrate buffer. Three days later, blood was collected from the tail vein, and the random blood glucose was measured. Rats with blood glucose levels ≥ 16.7 mmol/L were considered to have diabetes mellitus, and 36 rats were identified as diabetic rats. Ten rats in the NC group and 30 rats in the diabetes mellitus group were randomly selected for use in subsequent experiments. Then, 30 diabetic rats were randomly divided into the diabetic cataract group (DC, n = 10), the DMSO solvent group (DMSO, n = 10) and the rapamycin treatment group (RAPA, n = 10). Animals in the DC group received no extra treatment. Diabetic rats in the DMSO group received an intraperitoneal injection of an equal volume of DMSO, the rapamycin solvent, qod. Diabetic rats in the RAPA group received an intraperitoneal injection of rapamycin (2 mg/kg, qod).

### Cell culture and treatment

HLE-B3 cells were purchased from the American Type Culture Collection (Manassas, VA, USA) and were identified by Tsingke Biological Technology (Beijing, China) via short tandem repeat (STR) profiling. HLE-B3 cells were cultured in Dulbecco's modified Eagle’s medium (DMEM, 5.5 mM glucose, Hyclone, Logan, UT, USA) containing 10% fetal bovine serum and 1% penicillin-streptomycin and incubated at 37 °C with 5% CO_2_.

After reaching 70–80% confluence, HLE-B3 cells were divided into six groups and cultured for 24 h. Cells in the NC group received no treatment, while the cells cultured under high-glucose conditions were cultured with DMEM in addition with 30 mM glucose. The cells cultured under high-glucose conditions were then divided into the high glucose group (HG); the DMSO solvent group (DMSO), which was treated with DMSO (equal volume solvent of rapamycin and DAPT); the rapamycin treatment group (RAPA), which was treated with 200 nM rapamycin; the DAPT treatment group (DAPT), which was treated with 5 μM DAPT; and the combined treatment group (R + D), which was treated with both 200 nM rapamycin and 5 μM DAPT.

### Observation of lens opacity and grading

Lens opacity was observed before STZ treatment (0 w) and 4 weeks (4 w) and 8 weeks (8 w) after STZ treatment. Before the examination, the rats were anesthetized by administering intraperitoneal injection of 1% sodium pentobarbital (Sigma-Aldrich, St. Louis, MO, USA) solution (40 mg/kg). The degree of lens opacity was observed and photographed under a slit-lamp microscope (Carl Zeiss, Jena, Germany) after mydriasis with tropicamide (Sinqi, Shenyang, China).

The grade of lens opacity was determined using the method described by Shizuo Ao et al. (Ao et al. 1991): Grade 0, clear lens; Grade 1, peripheral vesicles; Grade 2, vesicles and cortical opacities; Grade 3, diffuse central opacities; and Grade 4, nuclear cataract.

### Preparation of lens paraffin sections and hematoxylin and eosin (H&E) staining

After the rats were sacrificed by administering an overdose of pentobarbital sodium, the eyeballs were removed and then transferred to ice-cold PBS buffer. The lens was gently dissected, and the tissues that adhered to the surface of the lens, such as the iris, ciliary body and suspensory ligament, were carefully removed under a stereomicroscope (Olympus, Tokyo, Japan).

Lenses were fixed with FAS eyeball fixation solution (Servicebio, Wuhan, China) at room temperature for more than 24 h, embedded in paraffin and cut into 4 μm sagittal slices. The sections were washed and incubated with hematoxylin for 10 min at room temperature. Then, they were rinsed with deionized water and dipped in a 1% eosin solution for 15 s. After rehydration in an alcohol gradient, the slices were cleared in xylene and mounted. The histological changes in the lenses were observed under a microscope and photographed (Nikon, Tokyo, Japan).

### Immunofluorescence staining

Whole-mount lens anterior capsules lined with LECs of rats and cultured HLE-B3 cells were fixed with 4% paraformaldehyde (PFA) for 30 min and blocked with 0.5% Triton X-100 and 1% BSA for 1 h at room temperature. Then, the capsules and the cultured cells were incubated with primary antibodies overnight at 4 °C, followed by an incubation with secondary antibodies for 2 h at room temperature (Table 1). Finally, the cell nuclei were stained with 4′,6-diamidino-2-phenylindole (DAPI). The capsules and the cultured cells were observed and photographed under a confocal scanning laser microscope (Olympus, Tokyo, Japan).

**Table 1:** Antibodies

Antibody	Host	Applications	Source	Catalog number
E-cadherin	Mouse	WB,IF	CST, USA	14,472
ZO-1	Rabbit	WB	CST, USA	8193
α-SMA	Rabbit	WB,IF	CST, USA	19,245
Fibronectin	Rabbit	WB	Abcam, UK	ab45688
Snail	Rabbit	WB	CST, USA	3879
LC3A/B	Rabbit	WB,IP	CST, USA	12,741
SQSTM1/p62	Mouse	WB,IP	Abcam, UK	ab56416
Akt	Rabbit	WB	CST, USA	4691
Phospho-Akt (Ser473)	Rabbit	WB	CST, USA	4060
mTOR	Rabbit	WB	CST, USA	2983
Phospho-mTOR (Ser2448)	Rabbit	WB	CST, USA	5536
ULK1	Rabbit	WB,IP,IF	CST, USA	8054
Phospho-ULK1 (Ser757)	Rabbit	WB	CST, USA	14,202
Jagged1	Rabbit	WB	CST, USA	70,109
Notch1	Rabbit	WB,IP	CST, USA	3608
Notch1	Mouse	IF	Santa Cruz, USA	sc-376403
NICD	Rabbit	WB,IP,IF	Abcam, UK	ab8925
Snail	Rabbit	WB	CST, USA	3879
Smad2/3	Rabbit	WB,IP	CST, USA	8685
GAPDH	Mouse	WB	Proteintech, China	60,004–1-Ig
Anti-mouse IgG (H + L)	Goat	WB	Proteintech, China	SA00001-1
Anti-rabbit IgG (H + L)	Goat	WB	Proteintech, China	SA00001-2
Anti-rabbit IgG (H + L) Alexa Fluor 594	Donkey	IF	Yeasen, China	34212ES60
Anti-mouse IgG (H + L) Alexa Fluor 594	Goat	IF	Yeasen, China	33212ES60
Anti-rabbit IgG (H + L) Alexa Fluor 488	Donkey	IF	Yeasen, China	34206ES60
Normal Rabbit IgG	Rabbit	IP	Sigma-Aldrich, USA	12–370
Normal Mouse IgG	Mouse	IP	Sigma-Aldrich, USA	12–371

### Quantification of autophagosomes

The rat lenses were fixed with glutaraldehyde (4%, Solarbio, Beijing, China) for 24 h. The anterior part of the lens with the lens epithelium and superficial cortex was cut into several small blocks of approximately 1 × 1 × 2 mm and then fixed for another 24 h. After dehydration in acetone and embedding in epoxy resin, ultrathin sections of the lens epithelium were cut and stained with uranyl acetate buffer and lead citrate. Then, the ultrastructure of the rat LECs was observed and photographed under a transmission electron microscope (TEM, Hitachi, Tokyo, Japan) at 10,000 × and 30,000 × magnification, and the number of autophagosomes in 5 random fields was counted at 10,000 × magnification for each sample, and three samples per group were evaluated.

HLE-B3 cells were harvested and centrifuged by high-speed cryogenic centrifugation (2000 rpm) at 4 °C for 5 min and then fixed with glutaraldehyde for 24 h. The steps used for this procedure were the same as those described above.

### Western blotting

Rat lens anterior capsules lined with LECs and cultured HLE-B3 cells were lysed with RIPA buffer (Beyotime, Shanghai, China). The protein concentration was determined using a BCA protein assay kit (Beyotime, Shanghai, China). Appropriate concentrations of the target proteins were separated on SDS-PAGE gels according to their molecular weights and then transferred to PVDF membranes (Millipore, Burlington, MA, USA). The proteins on the membrane were blocked with 5% skim milk for 2 h at room temperature and incubated overnight with the primary antibodies at 4 °C. Then, the proteins were incubated with HRP-conjugated secondary antibodies for 1 h at room temperature (Table 1) and visualized using enhanced chemiluminescence (ECL, 4A Biotech, Beijing, China). Proteins were quantified using ImageJ software (National Institutes of Health, USA). All experiments were repeated three times.

### Transwell assay

A 200 μL cell suspension (1 × 10^5^ cells/mL) was seeded in the upper chamber of transwell chambers (Millipore, Burlington, MA, USA), and 800 μL of the medium required for each group were added to the bottom chambers. The cells were incubated for 24 h. Then, the cells in the upper chamber were carefully removed, and the chambers were placed in 4% PFA for 30 min and stained with 0.1% crystal violet for 20 min. The stained cells in five random fields of each well were counted under an inverted microscope (Olympus, Tokyo, Japan).

### Scratch Wound Assay

HLE-B3 cells were seeded in 6-well plates, incubated for 24 h, and the cells in each well were scraped with a sterile micropipette tip (200 μL). After 3 washes with PBS, the cells were cultured in DMEM supplemented with different treatments according to their groups for 24 h. Images of each well were captured at 0 (0 h, original time) and 24 h (24 h). The wound area was marked and calculated using ImageJ software.

$${\text{Cell migration }}\left( \% \right)\, = \,({\text{original wound area at}}\,0 \,{\text{h}} - {\text{new wound area at}}\,{24}\, {\text{h}})/{\text{original wound area at}}\,0 \,{\text{h}}\, \times \,{1}00\% .$$

### Coimmunoprecipitation (Co-IP) assay

Cultured HLE-B3 cells were collected and incubated in IP lysis buffer (Beyotime, Shanghai, China) for 1 h. After centrifugation at 12,000 rpm for 10 min, the supernatant of the cell lysates was collected, 10 μL of the supernatant were used for protein quantification, and the remaining supernatant was incubated with specific antibodies or normal IgG from the same species with gentle rotation overnight at 4 °C (Table 1). After the sample was rinsed with IP lysis buffer, Protein A/G magnetic beads (Thermo Fisher Scientific, Waltham, USA) were added and incubated for 4 h. The beads were washed with PBS 3 times and then washed with IP lysis buffer 2 times. Finally, the precipitated proteins were eluted and denatured in 2 × SDS loading buffer (Beyotime, Shanghai, China) at 100 °C for 10 min and then analyzed using Western blotting.

### Cell transfection

The pEGFP-C3 and pEGFP-C3-NICD plasmids were synthesized by LaideerM Bio (Guangzhou, China). The pcDNA3.1-ULK1 (S758A) plasmid expressing constitutively active ULK1 with the serine residue at position 758 mutated to alanine, pcDNA3.1-ULK1 and pcDNA3.1 plasmids were generated by LaideerM Bio (Guangzhou, China). When HLE-B3 cells cultured in DMEM reached 70%–80% confluence, they were transfected with Lipofectamine 3000 (Invitrogen, Carlsbad, CA, USA) for 36 h.

### Statistical analysis

GraphPad Prism 7.0 and SPSS 24.0 were used for statistical analyses in this study. The Kruskal-Wallis H test with Bonferroni’s correction were used to compare ranked data. Measurement data are presented as the means ± standard deviations, and comparisons between multiple groups were performed using one-way analysis of variance (ANOVA) with Tukey’s post hoc test. *P* < 0.05 was considered a significant difference.

## Results

### Effects of rapamycin on body weight and random blood glucose levels in diabetic rats

**Fig. 1:**
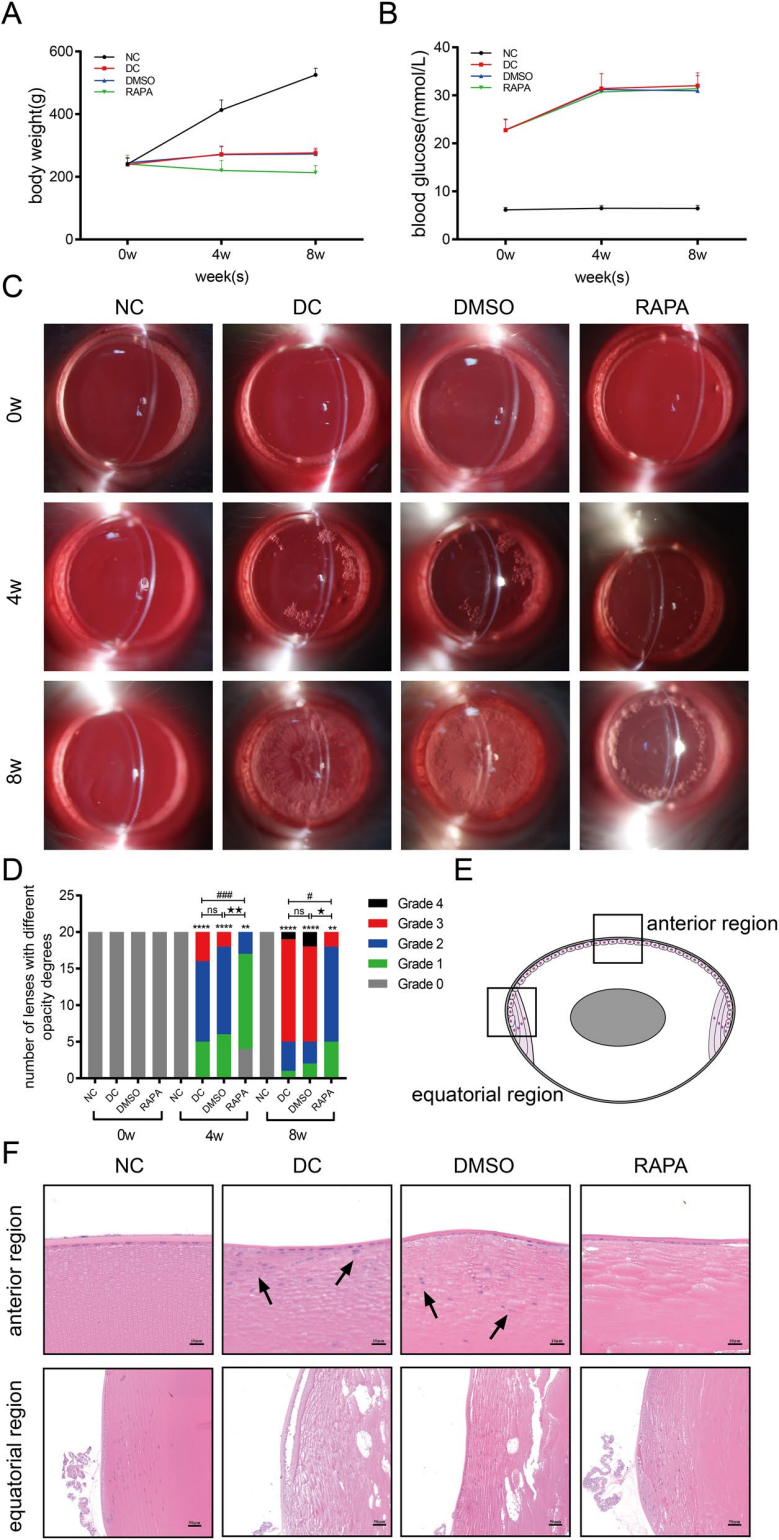
Rapamycin prevented the formation of cataracts in diabetic rats. Diabetic rats (DC) were induced by STZ. Rats in the normal control group (NC) were injected with the corresponding volume of citric acid-sodium citrate buffer. Diabetic rats were treated with DMSO solvent (DMSO) and rapamycin (RAPA) respectively. Body weight (**A**, n = 10) and random blood glucose levels (**B**, n = 10) of rats were examined after treatment with rapamycin for 0 (before treatment), 4 and 8 w. (**C**,** D**) Lenses of rats in each group (n = 20) were observed and photographed under a slit lamp microscope at 0 w, 4 w and 8 w after treatment with rapamycin. **E**, **F** H&E staining showed histological changes in the lens (n = 4). ns indicates *P* > 0.05; ^**^P < 0.01 and ^****^P < 0.0001 *vs.* NC group; ^#^*P* < 0.05 and ^###^*P* < 0.001 *vs.* DC group; ^★^*P* < 0.05 and ^★★^*P* < 0.01 *vs.* DMSO group

The body weight and random blood glucose levels of the rats were examined after treatment with rapamycin for 0 w (before treatment), 4 w and 8 w. The mean body weights of the three diabetic groups (DC, DMSO, and RAPA group) were obviously lower than that of the control group, and the body weight in the RAPA group was lower than that in the DC and DMSO groups (Fig. 1A). Before treatment with rapamycin (0 w), at 4 w and at 8 w, the random blood glucose levels measured in the three diabetic groups were higher than those in the normal group, while no significant differences were observed among the three diabetic groups (Fig. 1B).

### Rapamycin prevented cataract formation in diabetic rats

The lenses of rats in each group were observed and photographed under a slit lamp microscope at 0 w, 4 w, and 8 w (Fig. 1C, D). The lenses in the NC group remained transparent for 8 w. At 4 w, the lenses in the three diabetic groups showed various degrees of opacification. The degree of opacity of most lenses in the DC group (11/20) and the DMSO group (12/20) was Grade 2, the vacuoles at the equator of the lenses gradually expanded toward the center, and light cloud-like opacity appeared in the nuclear area of the lenses. In the RAPA group, the degree of opacity of most lenses was Grade 1, and vacuoles only appeared around the equator of the lenses. No significant difference in the degree of opacity was observed between the DC group and the DMSO group (adjusted *P* > 0.05), while the degree of lens opacity in the RAPA group was significantly lower than that in the DC group and the DMSO group (all adjusted *P* < 0.05). At 8 w, lens opacity in the three diabetic groups continued to increase. The degree of opacity of most lenses in the DC group (14/20) and the DMSO group (13/20) was Grade 3, and the vacuoles became denser and extended to the nuclear area. In the RAPA group, the degree of opacity of most lenses (13/20) was Grade 2. The statistical analysis revealed that the results were consistent with those obtained at 4 w. Based on these results, rapamycin delayed and prevented the development of cataracts in diabetic rats.

Histological changes in the lenses are showed in Fig. 1E, F. In the NC group, the LECs underneath the center anterior lens capsule were arranged in a single layer, and they showed a physiological bow arrangement at the equatorial region. The lens fibers in the superficial cortex displayed an orderly and tight arrangement. In the DC and DMSO groups, the lens epithelium was arranged in multiple layers, and some LECs migrated into the superficial cortex. Lens fibers in the cortex showed edema and vacuole formation. After treatment with rapamycin (RAPA group), the lens epithelium was basically maintained in a single flat structure, and the edema of lens fibers in the cortex was reduced.

### Rapamycin activated autophagy and inhibited EMT in the lens of diabetic rats

**Fig. 2:**
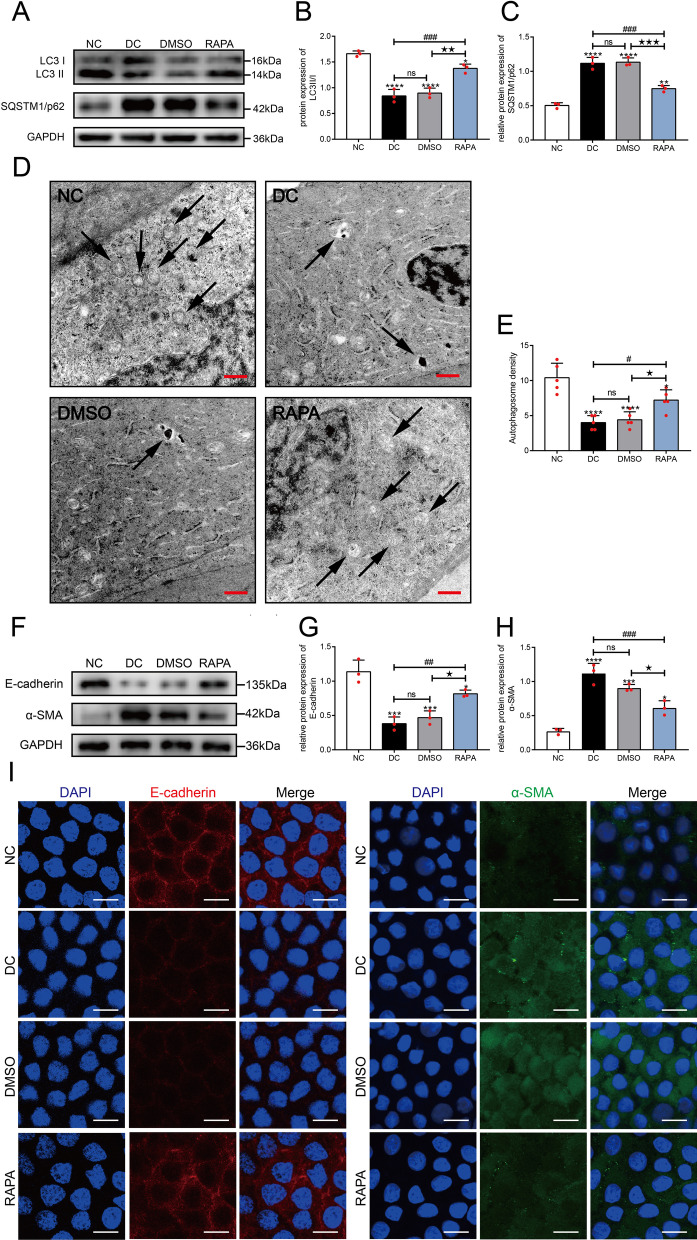
Rapamycin activated autophagy and inhibited the EMT in LECs from diabetic rats. Diabetic rats (DC) were induced by STZ. Rats in the normal control group (NC) were injected with the corresponding volume of citric acid-sodium citrate buffer. Diabetic rats were treated with DMSO solvent (DMSO) and rapamycin (RAPA) for 8 weeks respectively. Levels of the LC3 and SQSTM1/p62 proteins in the LECs of rats in each group were detected using Western blotting at 8 w (**A**). Statistical analysis of the relative expression of LC3II/I **B** and SQSTM1/p62 (**C**), n = 3. **D** Representative TEM images of autophagosomes (arrows) in different groups, bar = 0.5 µm. **E** Quantification and statistical analysis of the number of autophagosomes, n = 5. **F** E-cadherin and α-SMA protein levels in LECs from rats in each group were detected using Western blotting at 8 w. Statistical analysis of the relative expression of E-cadherin **G** and α-SMA (**H**), n = 3. **I** Immunofluorescence staining for E-cadherin (red) and α-SMA (green) in LECs from the different groups, n = 3. Bar = 10 µm. ns indicates *P* > 0.05; ^*^P < 0.05, ^**^P < 0.01, ^***^P < 0.001 and ^****^P < 0.0001 *vs.* NC group; ^#^*P* < 0.05, ^##^*P* < 0.01, ^###^*P* < 0.001 *vs.* DC group; ^★^*P* < 0.05, ^★★^*P* < 0.01 and ^★★★^*P* < 0.001 *vs.* DMSO group

At 8 w, the proteins in the LECs from the rats in each group were extracted, and autophagy marker proteins were detected using Western blot assays (Fig. 2A-C). Compared with the NC group, the expression of LC3 II/I in the LECs of the DC group was significantly reduced (*P* < 0.0001), while the expression of SQSTM1/p62 was increased (*P* < 0.0001). The expression levels of LC3 II/I and SQSTM1/p62 were not significantly different between the DC and DMSO groups (*P* > 0.05), indicating that autophagic activity in the lens epithelium of diabetic rats was inhibited. After treatment with rapamycin, the expression of LC3 II/I increased in the RAPA group (*P* < 0.0001) and the expression of SQSTM1/p62 decreased compared with that in both the DC group and the DMSO group (all *P* < 0.001), but the RAPA and NC groups showed significant differences in the expression of LC3II/I and SQSTM1/p62 (all *P* < 0.01).

Autophagosomes were observed under a TEM to further detect the changes in autophagic activity in the LECs of diabetic rats (Fig. 2D, E). The number of autophagosomes in the DC group was significantly reduced compared with that in the NC group (*P* < 0.0001). However, the number of autophagosomes increased in the RAPA group. Collectively, this evidence suggested that rapamycin partially rescued the reduced autophagic activity in the LECs of diabetic rats.

EMT marker proteins were detected using Western blot assays (Fig. 2F–H). The expression of E-cadherin was significantly downregulated (*P* < 0.001) and the expression of α-SMA was significantly upregulated (*P* < 0.001) in the DC group compared with the NC group. In addition, the expression of E-cadherin and α-SMA showed no differences between the DC and DMSO groups (*P* < 0.05), indicating that the EMT occurred in the lens epithelium of diabetic rats. Compared with the DC and DMSO groups, the expression of E-cadherin increased (all *P* < 0.05) and the expression of α-SMA decreased (all *P* < 0.05) in the RAPA group, suggesting that rapamycin not only increased autophagic activity but also partially prevented the EMT in the lens epithelium of diabetic rats. Alterations in both E-cadherin and α-SMA expression were also detected in whole-mount lens epithelium using immunofluorescence staining, and the results were consistent with those from the Western blot assays (Fig. 2I).

Therefore, autophagy was suppressed but the EMT was activated in the lens epithelium of diabetic rats. The EMT was effectively prevented by enhancing autophagic activity with rapamycin, resulting in delayed formation of diabetic cataracts.

### Rapamycin activated autophagy by inhibiting the mTOR/ULK1 signaling pathway in high-glucose-stimulated HLE-B3 Cells

**Fig. 3:**
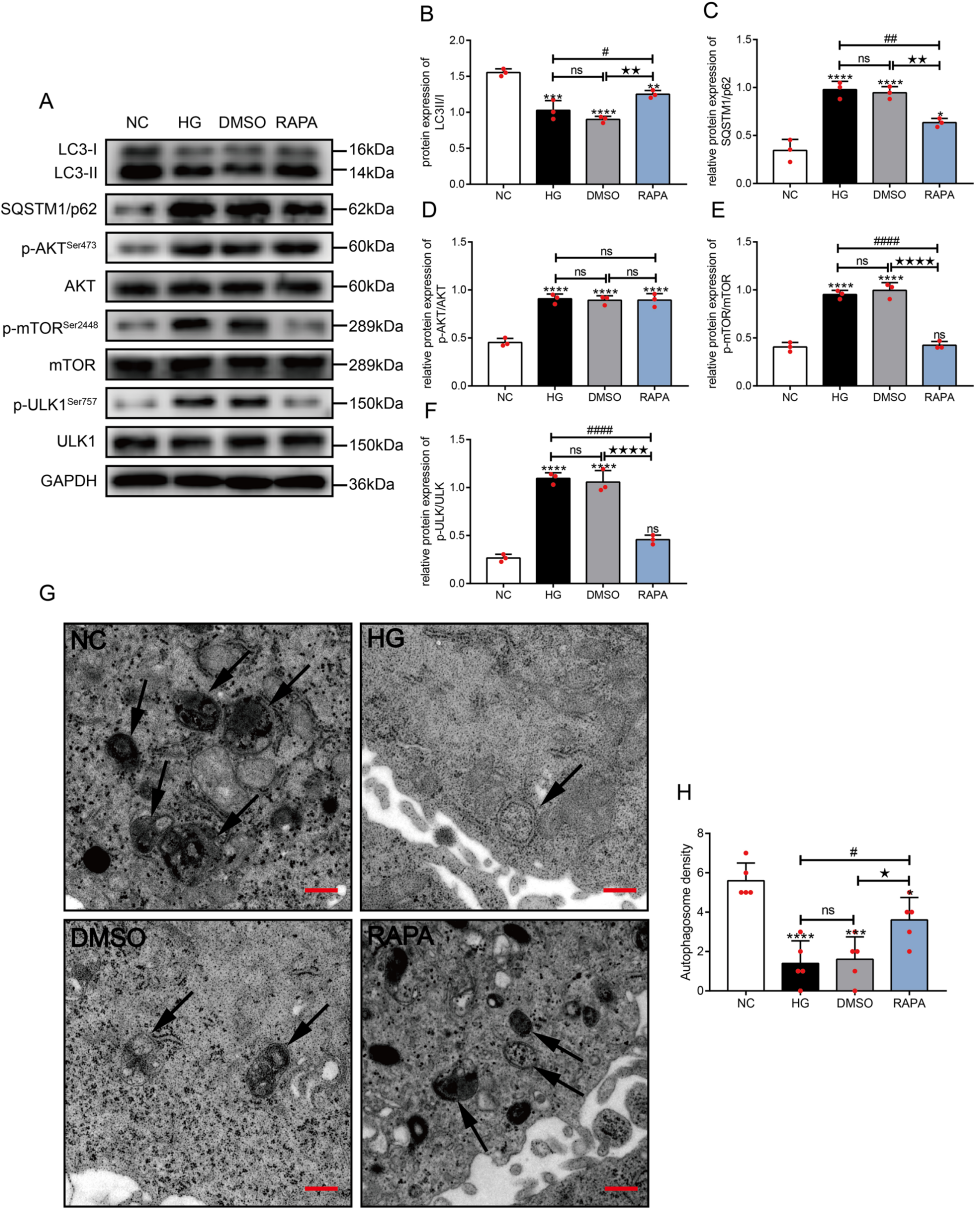
Rapamycin activated autophagy by inhibiting the mTOR/ULK1 signaling pathway in HLE-B3 cells cultured under high-glucose conditions. Cells were treated with high glucose (HG, 30 mM glucose), high glucose + DMSO (equal volume solvent of rapamycin) and high glucose + 200 nM rapamycin (RAPA) for 24 h, respectively. **A** The expression of molecules in the autophagy signaling pathway and autophagy marker proteins. Statistical analysis of the relative levels of LC3II/I **B** and SQSTM1/p62 (**C**), p-AKT/AKT (**D**), p-mTOR/mTOR **E** and p-ULK1/ULK1 (**F**), n = 3.** G** Representative TEM images of autophagosomes (arrows) in different groups. **H** Quantification and statistical analysis of the number of autophagosomes, n = 5. ns indicates *P* > 0.05; ^*^P < 0.05, **P < 0.01, ***P < 0.001 and ****P < 0.0001 *vs.* NC group; ^#^*P* < 0.05, ^##^*P* < 0.01, ^###^*P* < 0.001 and ^####^*P* < 0.0001 *vs.* HG ^group; ★*P* < 0.05, ★★*P* < 0.01, ★★★*P* < 0.001 and ★★★★*P* < 0.0001^ *vs.* DMSO group

We conducted additional in vitro experiments to explore whether activation of autophagy regulated the EMT of LECs under high-glucose conditions. HLE-B3 cells were stimulated with high glucose concentrations and treated with rapamycin. The expression of LC3 II/I was decreased (*P* < 0.001) and SQSTM1/p62 expression was increased (*P* < 0.0001) after stimulation with high glucose concentrations compared with those in the NC cells. After treatment with rapamycin, the expression of LC3 II/I was increased (*P* < 0.001) and SQSTM1/p62 expression was decreased (*P* < 0.0001) compared with the levels detected in cells cultured under high-glucose conditions (Fig. 3A–C), but these parameters still differed from those in the NC group. Under TEM, the number of autophagosomes in HLE-B3 cells in the HG group was significantly reduced compared with the number in the NC group (*P* < 0.0001). However, the number of autophagosomes increased in the RAPA group (Fig. 3G, H). Altered protein expression in the signaling pathway regulating autophagic activity in HLE-B3 cells was also observed using Western blot assays. The levels of p-AKT, p-mTOR, and p-ULK1 were all increased (all *P* < 0.0001) in HLE-B3 cells cultured under high-glucose conditions (in both the HG and DMSO groups) compared with those in NC cells, but the expression of AKT, mTOR, and ULK1 was not significantly altered (*P* > 0.05). After treatment with rapamycin, the levels of both p-AKT and AKT (*P* > 0.05), mTOR, and ULK1 were not significantly changed (*P* > 0.05), whereas the levels of p-mTOR and p-ULK1 were significantly decreased (*P* < 0.0001) compared with those in cells cultured under high-glucose conditions, and they were not significantly different from those in the NC group (Fig. 3A, D–F). Therefore, rapamycin activated autophagy by inhibiting the mTOR/ULK1 signaling pathway.

In summary, autophagy was inhibited in HLE-B3 cells cultured under high-glucose conditions, and rapamycin enhanced autophagic activity through the mTOR/ULK1 signaling pathway.

### Rapamycin inhibited the EMT of HLE-B3 cells treated with high glucose concentrations through the notch signaling pathway

**Fig. 4:**
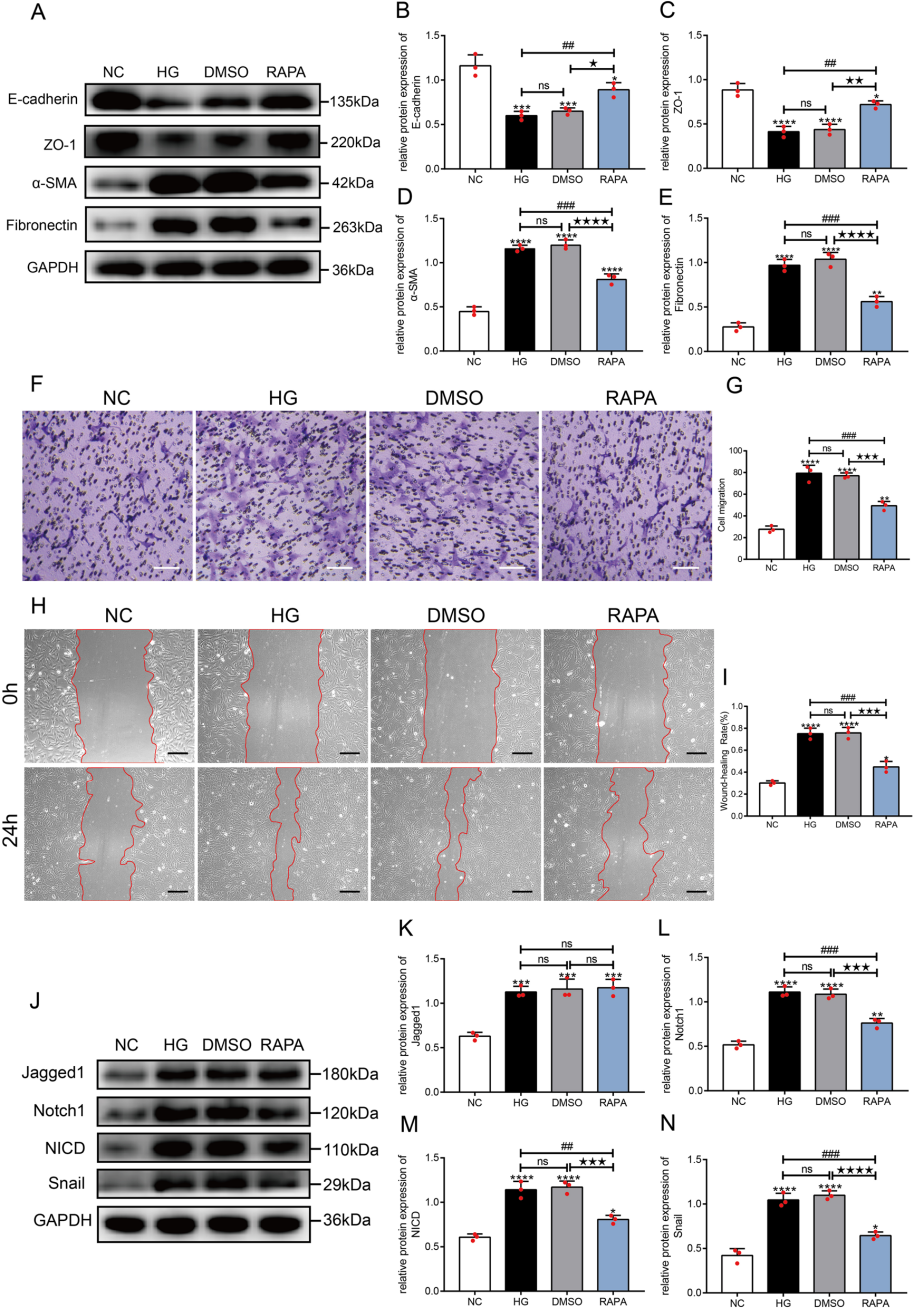
Rapamycin inhibited the EMT of HLE-B3 cells cultured under high-glucose conditions by modulating the Notch signaling pathway. HLE-B3 cells were stimulated with 30 mM glucose (HG group), and then treated with DMSO (DMSO group, equal volume solvent of rapamycin), 200 nM rapamycin (RAPA group) for 24 h, respectively. **A** The expression of E-cadherin, ZO-1, α-SMA and Fibronectin in HLE-B3 cells from each group was detected using Western blotting at 24 h. Statistical analysis of the relative expression of E-cadherin (**B**), ZO-1 (**C**), α-SMA **D** and Fibronectin (**E**), n = 3. Cell migration after exposure to different treatments for 24 h was detected by performing a transwell assay and scratch wound assay. Representative images and the quantification of the transwell assay (**F**, **G**, n = 3, bar = 100 μm) and scratch wound assay (**H**, **I**, n = 3, bar = 200 μm). **J** Expression of proteins in the Notch signaling pathway in HLE-B3 cells at 24 h. Quantification of the relative levels of Jagged1 (**K**), Notch1 (**L**), NICD (**M**) and Snail (**N**), n = 3. ns indicates *P* > 0.05; ^*^P < 0.05, ^**^P < 0.01, ^***^P < 0.001 and ^****^P < 0.0001 *vs.* NC group; ^##^*P* < 0.01 and ^###^*P* < 0.001 *vs.* HG group; ^★^*P* < 0.05, ^★★★^*P* < 0.001 and ^★★★★^*P* < 0.0001 *vs.* DMSO group

HLE-B3 cells were stimulated with high glucose concentrations and treated with rapamycin. The expression levels of E-cadherin and ZO-1 were downregulated (*P* < 0.001) and α-SMA and Fibronectin were significantly upregulated (*P* < 0.0001) in the HG group compared with those in the NC group. After treatment with rapamycin (RAPA group), the expression levels of E-cadherin and ZO-1 were increased (*P* < 0.05), and α-SMA and Fibronectin expression levels were significantly decreased (*P* < 0.05) compared with those in the high glucose groups (HG and DMSO groups), yet the significant differences were still observed between the RAPA group and NC group (all *P* < 0.05) (Fig. 4A–E).

Cell migration was determined by performing transwell and scratch wound assays. Under high-glucose conditions, cell migration was significantly increased compared with that of normal cultured cells (all *P* < 0.0001). After treatment with rapamycin, migration was inhibited compared with that in the high-glucose and DMSO-treated cells, suggesting that rapamycin partially restrained the EMT of HLE-B3 cells exposed to high glucose concentrations (Fig. 4F–I).

The Notch signaling pathway consisting of Jagged1/Notch1/NICD/Snail is a classic signaling pathway regulating the EMT and is activated by TGFβ2 during the EMT of LECs (Han et al. 2019; Chen et al. 2017). Western blot assays (Fig. 4J–N) showed higher levels of Jagged1, Notch1, NICD and Snail in the HG and DMSO groups than those in the NC group (all *P* < 0.001). Compared with the HG and DMSO groups, Jagged1 expression was not significantly altered after treatment with rapamycin (*P* > 0.05), but the expression levels of Notch1, NICD and Snail were significantly decreased (all *P* < 0.001), although they were still higher than those in the NC group (all *P* < 0.05). Thus, the activation of autophagy partially blocked the activation of Notch signaling pathway stimulated by high glucose concentrations.

**Fig. 5:**
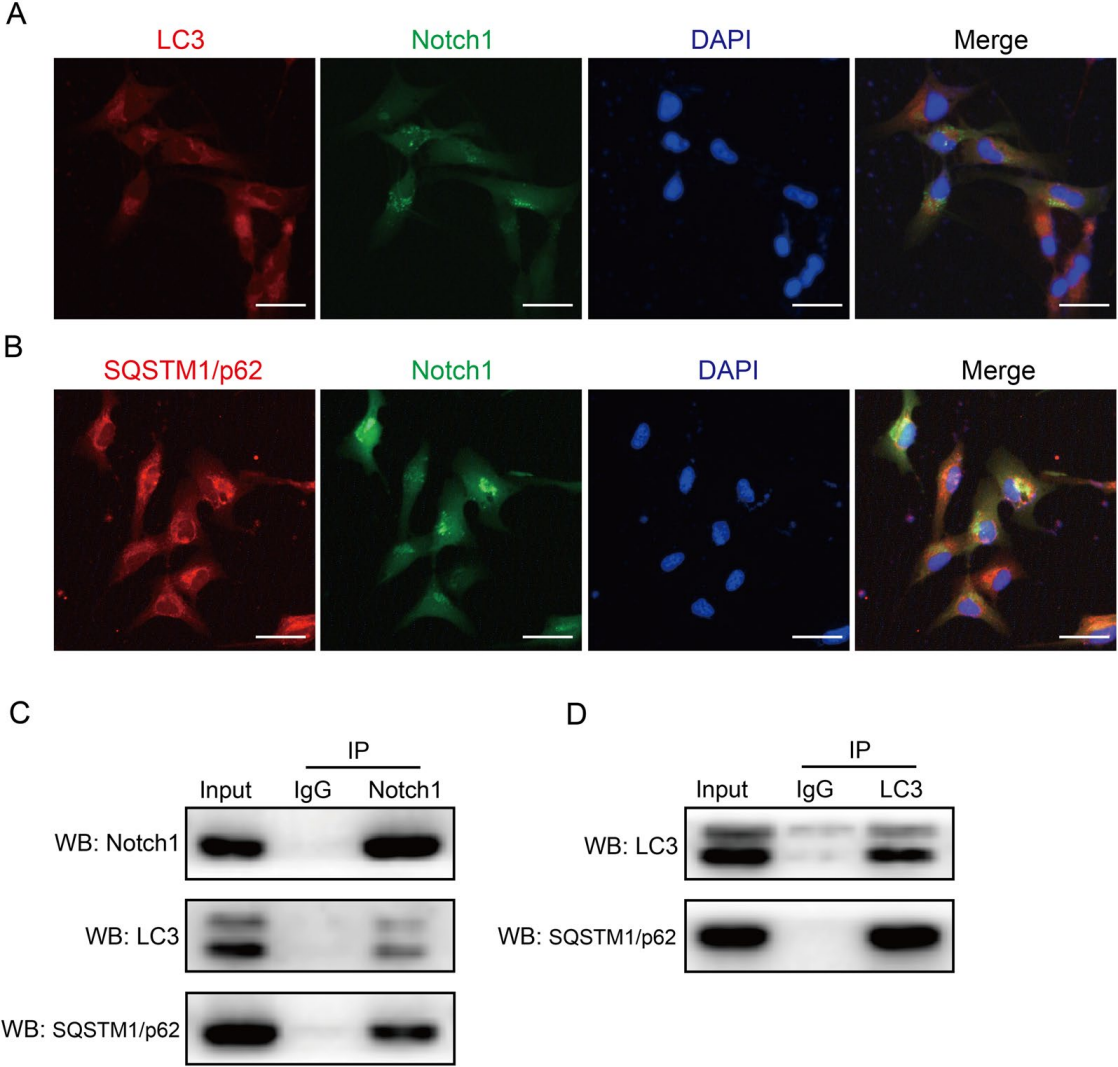
Autophagy promoted Notch1 degradation through the interactions of Notch1, LC3 and SQSTM1/p62. HLE-B3 cells were cultured in DMEM (5.5 mM glucose) for 24 h.** A** Immunofluorescence staining for LC3 (red) and Notch1 (green) in HLE-B3 cells, bar = 40 μm.** B** Immunofluorescence staining for SQSTM1/p62 (red) and Notch1 (green) in HLE-B3 cells, bar = 40 μm.** C**,** D** Co-IP of Notch1, LC3 and SQSTM1/p62. IP: immunoprecipitation, WB: Western blot, n = 3

Immunofluorescence staining showed the colocalization of Notch1 and LC3 (Fig. 5A) and of Notch1 and SQSTM1/p62 (Fig. 5B) in the cytoplasm of HLE-B3 cells. Co-IP showed that Notch1 interacted with LC3 and SQSTM1/p62 (Fig. 5C), and LC3 interacted with SQSTM1/p62 (Fig. 5D). Based on these results, autophagy might mediate the selective degradation of Notch1 through interactions with LC3 and SQSTM1/p62, thus negatively regulating the Notch signaling pathway.

### Crosstalk between the EMT and autophagy signaling pathways

We employed not only rapamycin but also DAPT, a γ-secretase inhibitor that blocks the final step of Notch cleavage and activation and then inhibits the Notch signaling pathway in vivo and in vitro (Park et al. 2015; Jiao et al. 2014), to evaluate the crosstalk between autophagy signaling pathways mediated by the mTOR/ULK1 and the EMT signaling pathways mediated by Jagged1/Notch1/NICD/Snail in HLE-B3 cells.

**Fig. 6:**
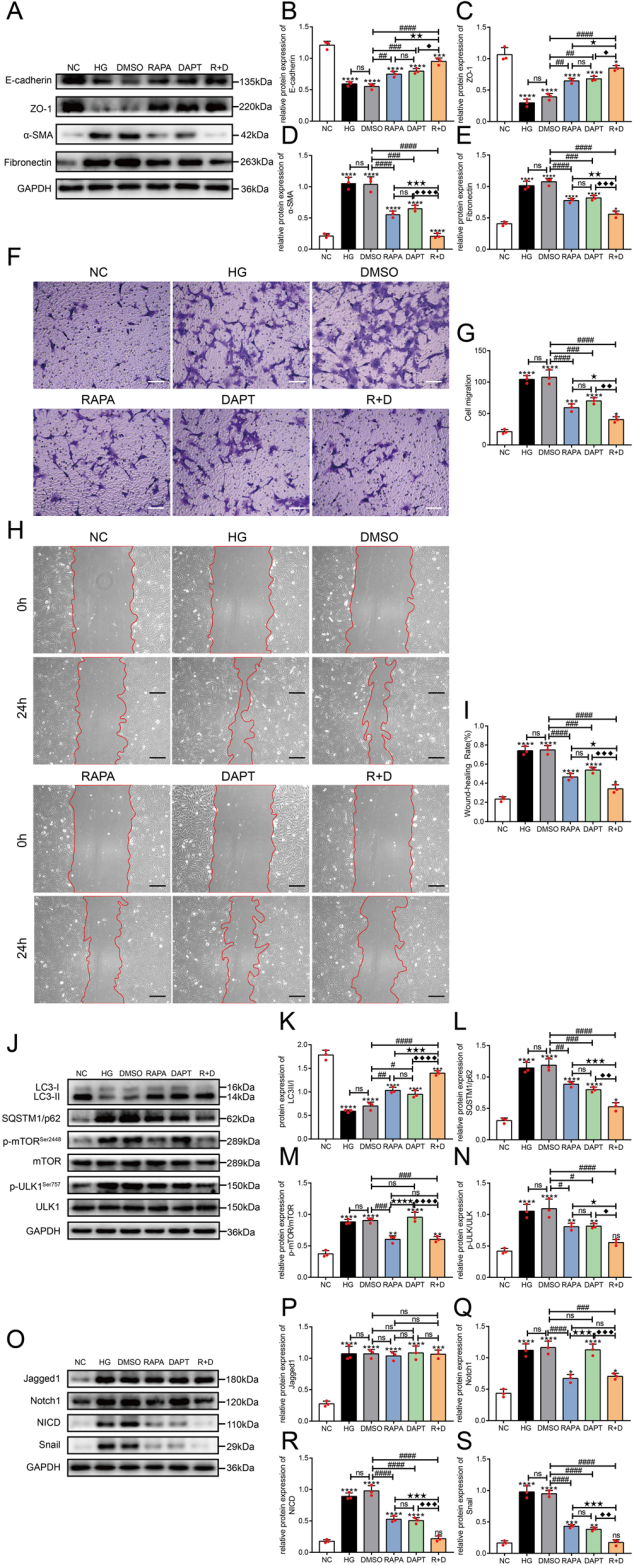
Effects of rapamycin combined with DAPT on autophagy and the EMT in HLE-B3 cells cultured under high-glucose conditions.. HLE-B3 cells were stimulated with 30 mM glucose (HG), then treated with DMSO solvent (DMSO), 200 nM rapamycin (RAPA), 5 μM DAPT (DAPT), and combination of 200 nM rapamycin with 5 μM DAPT (R + D) for 24 h, respectively. **A** The expression of E-cadherin, ZO-1, α-SMA and Fibronectin in HLE-B3 cells from each group was detected using Western blotting at 24 h. Statistical analysis of the relative expression of E-cadherin (**B**), ZO-1 (**C**), α-SMA **D** and Fibronectin (**E**), n = 3. Cell migration after exposure to different treatments for 24 h was detected by performing a transwell assay and scratch wound assay. Representative images and results of the transwell assay (**F**, **G**, n = 3, bar = 100 μm) and scratch wound assay (**H**, **I**, n = 3, bar = 200 μm). **J** The expression of molecules in the autophagy signaling pathway and autophagy marker proteins. Statistical analysis of the relative levels of LC3II/I (**K**), SQSTM1/p62 (**L**), p-mTOR/mTOR (**M**) and p-ULK1/ULK1 (**N**), n = 3. **O** The expression of proteins in the Notch signaling pathway. Quantification of the relative levels of Jagged1 (**P**), Notch1 (**Q**), NICD (**R**) and Snail (**S**), n = 3. ns indicates *P* > 0.05; *P < 0.05, **P < 0.01, ***P < 0.001 and ^****^P < 0.0001 *vs.* NC group; ^#^*P* < 0.05, ^##^*P* < 0.01, ^###^*P* < 0.001 and ^####^*P* < 0.0001 *vs.* DMSO group; ^★^*P* < 0.05, ^★★^*P* < 0.01, ^★★★^*P* < 0.001 and ^★★★★^*P* < 0.0001 *vs.* RAPA group; ^◆^*P* < 0.05, ^◆◆^*P* < 0.01, ^◆◆◆^*P* < 0.001 and ^◆◆◆◆^*P* < 0.0001 *vs.* DAPT group

Western blot assays showed no significant differences in the expression of E-cadherin, ZO-1, α-SMA and Fibronectin between the DAPT and RAPA groups, although the levels were significantly different from those in the high-glucose groups (HG and DMSO groups). However, after combined treatment with rapamycin and DAPT (R + D group), the alterations in the levels of EMT marker proteins were more significant than those in the groups treated with RAPA and DAPT alone (Fig. 6A–E). In addition, transwell and scratch wound assays revealed that either rapamycin or DAPT effectively inhibited cell migration induced by high glucose concentrations, and the combination of rapamycin and DAPT prevented cell migration much more significantly than either of treatment alone (Fig. 6F–I).

The detection of the expression of LC3 and SQSTM1/p62 proteins showed that either DAPT or rapamycin significantly increased autophagic activity in HLE-B3 cells stimulated with high glucose concentrations, and treatment with the combination of rapamycin and DAPT exerted a stronger effect than either compound alone (Fig. 6J–L). Among the proteins that regulate autophagy, p-mTOR, mTOR, p-ULK1 and ULK1 were assessed. Rapamycin effectively decreased the levels of p-mTOR and p-ULK1, while DAPT had no obvious effects on the levels of p-mTOR but significantly reduced the levels of p-ULK1 compared with those in the HLE-B3 cells cultured under high-glucose conditions (HG and DMSO groups). After treatment with the combination of rapamycin and DAPT, p-mTOR levels were not decreased further than those observed after rapamycin treatment, but p-ULK1 levels showed a greater reduction (Fig. 6J, M, N). These results suggested that DAPT regulated the autophagic activity of HLE-B3 cells by modulating the phosphorylation of ULK1 at Ser757. As a γ-secretase inhibitor, the NICD level was reduced after DAPT treatment in HLE-B3 cells. We speculated that NICD regulated the phosphorylation of ULK1 at Ser757.

Among the key proteins in the Notch signaling pathway, Jagged1, Notch1, NICD and Snail were all upregulated in HLE-B3 cells cultured under high-glucose conditions. As expected, DAPT reduced the levels of NICD and Snail but had no significant effects on the expression of Notch1, while rapamycin significantly reduced the level of Notch1, NICD and Snail. After treatment with the combination of rapamycin with DAPT, the levels of NICD and Snail were reduced to a greater extent than those detected in the groups treated with each compound alone, but Notch1 expression was not reduced to a greater extent than that in the RAPA group (Fig. 6O–S).

Thus, the activation of autophagy by rapamycin inhibited the EMT through the Notch1/NICD/Snail signaling pathway, while the inhibition of the EMT with DAPT inhibited the NICD/Snail signaling pathway and simultaneously activated autophagy by decreasing p-ULK1 levels. Therefore, the crosstalk between the EMT and the autophagy signaling pathways was mediated by NICD signaling to p-ULK1.

### Enhanced interaction of NICD and ULK1 in HLE-B3 cells stimulated with high glucose concentrations

**Fig. 7:**
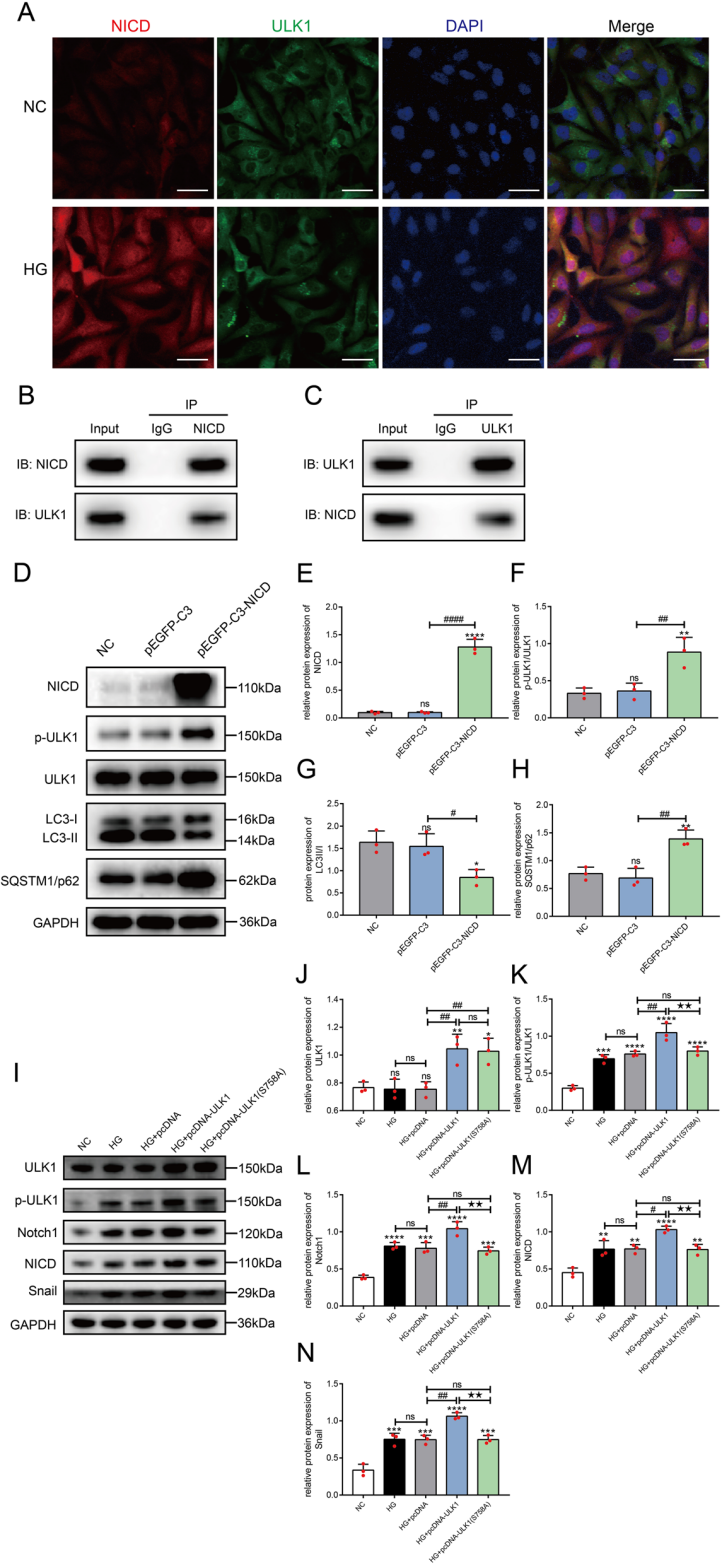
The Notch signaling pathway regulated autophagic activity through NICD-induced phosphorylation of ULK1. **A** Immunofluorescence staining for NICD (red) and ULK1 (green) in HLE-B3 cells cultured under normal-glucose (NC) and high-glucose conditions (HG), bar = 40 μm. **B**, **C** Co-IP of NICD and ULK1 in normal cultured HLE-B3 cells. *IP* immunoprecipitation, *WB* Western blot. **D** Verification of NICD overexpression plasmids, and detection of the levels of autophagy marker proteins and p-ULK1 after transfected with pEGFP-C3 and pEGFP-C3-NICD plasmids for 36 h in normal cultured HLE-B3 cells (NC) by Western blotting. Quantification of the relative levels of NICD (**E**), p-ULK1/ULK1 (**F**), LC3II/I **G** and SQSTM1/p62 (**H**), n = 3. ns indicates *P* > 0.05; *P < 0.05, **P < 0.01 and ****P < 0.0001 *vs.* NC group; ^#^*P* < 0.05, ^##^*P* < 0.01 and ^####^*P* < 0.0001 *vs.* HG + pEGFP-C3 group. **I** Verification of ULK1 and ULK1 (Ser 757) overexpression plasmids, and detection of the levels of proteins in the Notch signaling pathway and p-ULK1 after transfected with pcDNA3.1, pcDNA3.1-ULK1, and pcDNA3.1-ULK1 (S758A) plasmids for 36 h, respectively, in HLE-B3 cells cultured in high-glucose conditions (HG) by Western blot assays. Quantification of the relative levels of ULK1 (**J**), p-ULK1/ULK1 (**K**), Notch1 (**L**), NICD **M** and Snail (**N**), n = 3. ns indicates *P* > 0.05; ^*^P < 0.05, ^**^P < 0.01, ^***^P < 0.001 and ^****^P < 0.0001 *vs.* NC group; ^#^*P* < 0.05, ^##^*P* < 0.01 *vs.* HG + pcDNA group; ^★★^*P* < 0.01 *vs.* HG + pcDNA-ULK1 group

We found that DAPT inhibited the release of NICD and then prevented the phosphorylation of ULK1 stimulated by high glucose, thereby activating autophagy. Immunofluorescence staining showed that NICD and ULK1 colocalized in the cytoplasm of HLE-B3 cells, and this colocalization increased significantly after stimulation with high glucose concentrations. Moreover, NICD levels were increased and entered the nucleus (Fig. 7A). Co-IP assays proved an interaction between NICD and ULK1 in HLE-B3 cells stimulated with high glucose concentrations (Fig. 7B, C).

We overexpressed NICD by transfecting pEGFP-C3-NICD into HLE-B3 cells to further confirm that ULK1 phosphorylation is regulated by NICD, which resulted in increased levels of p-ULK1 and SQSTM1/p62 and the downregulation of LC3II/I expression (Fig. 7D–H), indicating the inhibition of autophagy.

Then, we overexpressed ULK1 and the ULK1 mutant at S758A using the pcDNA3.1 vector to verify the role of ULK1 phosphorylation in the Notch signaling pathway. The transfection was confirmed by performing Western blot assays. A significant difference in ULK1 expression was not observed between these two ULK1 overexpression groups; however, ULK1 phosphorylation at Ser 757 was significantly higher in the HG + pcDNA-ULK1 group than in the HG + pcDNA-ULK1 (S758A) group (Fig. 7I–K). Overexpression of ULK1, but not ULK (S758A), significantly increased the expression of proteins in the Notch pathway under high-glucose conditions (Fig. 7I, L–N), indicating that the phosphorylation of ULK1 at Ser 757 activated the Notch signaling pathway in HLE-B3 cells under high-glucose conditions.

Thus, the Notch signaling pathway was not activated in normally cultured HLE-B3 cells, as the amount of activated NICD in the cytoplasm was very low, its effects on ULK1 were weak, and autophagy proceeded normally. Under high-glucose conditions, the Notch signaling pathway was activated, and the NICD level in the cytoplasm increased substantially. The interaction of NICD and ULK1 increased the phosphorylation of ULK1 and inhibited the activity of ULK1, thereby blocking the initiation of autophagy and inhibiting the degradation of Notch1 by autophagy.

## Discussion

In the present study, we found that autophagy was inhibited and the EMT was activated in the LECs of diabetic cataracts and HLE-B3 cells under high-glucose conditions. We identified a new potential interaction in which NICD acts on ULK1, bridging the Jagged1/Notch1/NICD/Snail signaling pathway that activates the EMT and the mTOR/ULK1 signaling pathway that inhibits autophagy in LECs under high-glucose conditions. Activating autophagy with rapamycin inhibits the EMT of LECs by enhancing the degradation of Notch1 and delays the formation of diabetic cataracts. In turn, inhibiting the Notch signaling pathway with DAPT and reducing NICD levels in the cytoplasm reduces the interaction between NICD and ULK1, decreases the levels of p-ULK1, and then activates autophagy.

The correlation between the EMT of LECs and diabetic cataracts has been reported (Zhang et al. 2017; Du et al. 2017; Liu et al. 2020). We have also confirmed that high-glucose stimulation induces the EMT of LECs in vitro (Han et al. 2018; Li et al. 2020; Ye et al. 2020). In the present study, we have further verified that LECs undergo the EMT under high-glucose conditions both in vivo and in vitro. In recent years, researchers have focused on the role of the Notch signaling pathway in the development of cataracts. Chen et al. (Chen et al. 2017) have found that the Jagged-1/Notch signaling pathway is activated in the EMT of LECs induced by TGFβ2, and blocking Notch signaling reverses the EMT and lens fibrosis. Similarly, Han et al. (Han et al. 2019) have showed that miR-34a negatively regulates the EMT of LECs by targeting Notch1. In the present study, the Notch signaling pathway was activated in HLE-B3 cells cultured under high-glucose conditions and then induced the EMT.

Autophagy is essential for maintaining the homeostasis of cells, tissues and organs (Dikic and Elazar 2018), especially in the eye, including the cornea, retina, and lens (Chai et al. 2016). Impairment of autophagy flux in LECs and fiber cells may result in the formation of congenital cataracts, age-related cataracts, and diabetic cataracts (Morishita et al. 2013; Liu et al. 2020; Andley and Goldman 2016). In the present study, autophagic activity was attenuated both in LECs of diabetic rats and in HLE-B3 cells stimulated with high glucose concentrations, consistent with the findings of many other studies (Chen et al. 2021; Li et al. 2020). However, Liu has showed that autophagy changes from activation at the early stage to subsequent inhibition in the LECs of mice under high-glucose conditions (Liu et al. 2020). The possible explanations for this discrepancy are described below. First, the basic autophagic activity in the LECs of rats and mice may be different. Second, due to the faster development of diabetic cataracts in rats than in mice induced by STZ, we may only observe the final state of autophagy in LECs of diabetic rats and not the dynamic state during the development of cataracts.

The correlation or crosstalk between autophagy and the EMT has been explored in recent years. On the one hand, autophagy is required to maintain cell survival during the EMT. However, autophagy blocks the EMT and prevents cells from acquiring a mesenchymal phenotype (Chen et al. 2019). Due to the dual roles of autophagy in EMT, interfering with the EMT by targeting autophagy has become a new research direction. A large body of literature has showed that diabetic complications of the renal epithelium might be improved by increasing autophagic activity (Wang et al. 2019, 2021). Our study indicates that activation of autophagy with rapamycin inhibits the EMT of LECs in vivo and in vitro and then delays the development of cataracts.

Autophagy regulates Notch1 degradation during the development of stem cells and neurogenesis (Wu et al. 2016) and during the self-renewal of glioma-initiating cells and tumorigenicity (Tao et al. 2018). In addition, autophagy facilitates cardiac differentiation by degrading NICD (Jia et al. 2014). In addition to selective autophagy mediated by SQSTM1/p62 (Li et al. 2020), autophagy degrades proteins by promoting interactions with LC3 (Shvets et al. 2008). Our study suggests that rapamycin inhibits the EMT by increasing autophagic activity and then promotes the degradation of Notch1 through both the interaction of LC3 and SQSTM1/p62 with Notch1.

Inhibition of the Notch signaling pathway may exert different effects on autophagic activity. Natsumeda et al. have showed that blocking Notch signaling with MRK003, an inhibitor of γ-secretase, induces protective autophagy in glioblastoma neurospheres (Natsumeda et al. 2016). However, according to Zhang et al., inhibition of Notch signaling substantially diminishes autophagy (Zhang et al. 2017b). Interestingly, we found that DAPT, another inhibitor of γ-secretase, not only effectively inhibited the EMT but also activated autophagy in HLE-B3 cells under high-glucose conditions by reducing NICD levels. In murine T regulatory cells (Tregs), NICD regulates the autophagic activity by interacting with Beclin1 and Atg14 in the class III PI3K complex (Marcel and Sarin 2016), which is activated by the phosphorylation of the ULK1 complex (Dikic and Elazar 2018). The interaction between NICD and ULK1 has been verified through immunofluorescence assays and Co-IP assays, and solid evidence that overexpression of NICD phosphorylates ULK1 and then inhibits autophagy confirmed this interaction. The Notch signaling pathway is activated in HLE-B3 cells exposed to high glucose concentrations, and NICD levels are increased in the cytoplasm; thus, the interaction of NICD and ULK1 may prevent the activation of ULK1 by increasing its phosphorylation, thereby blocking the initiation of autophagy and finally inhibiting autophagic activity. Therefore, the efficacy of preventing the EMT and promoting autophagy is enhanced when rapamycin and DAPT are administered in combination.

Our study has some limitations. Regarding autophagic activity in LECs of diabetic rats, we might have only observed one state of autophagy in the formation of cataracts. Thus, we may need to observe the dynamic changes in autophagic activity during the development of cataracts. The effect of rapamycin in combination with DAPT on diabetic cataracts has not been verified in vivo. These questions will be answered in our subsequent studies.

## Conclusions

**Fig. 8:**
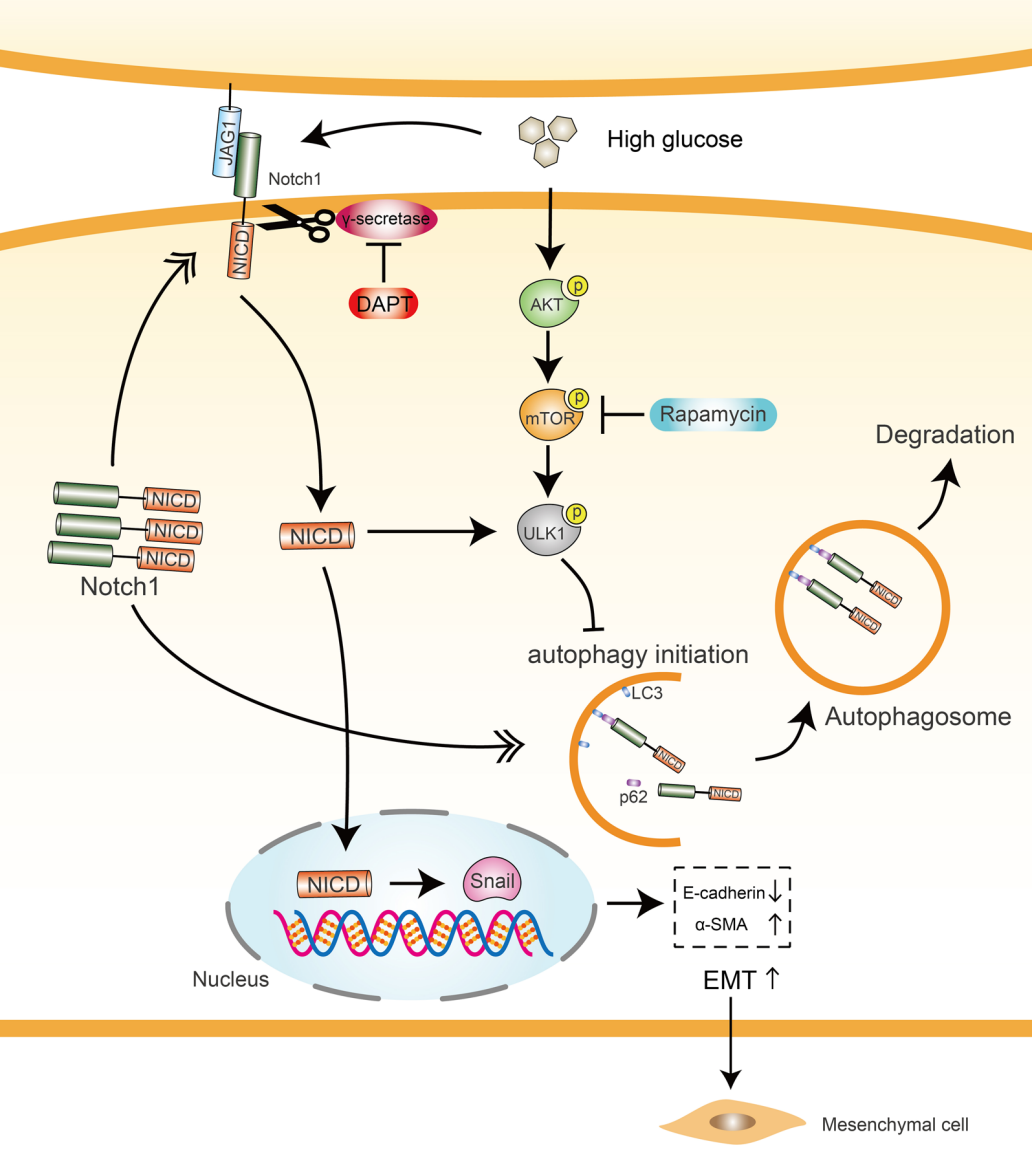
Schematic representation of the mechanism of mutual regulation between autophagy and the EMT in LECs stimulated with high glucose concentrations

In summary, we have showed that a high glucose concentration induces the EMT through the Jagged1/Notch1/NICD/Snail signaling pathway and inhibits autophagy through the AKT/mTOR/ULK1 pathway in LECs, and both processes may participate in the development of diabetic cataracts. As autophagic activity is reduced under high-glucose conditions, the selective degradation of Notch1 mediated by both SQSTM1/p62 and LC3 decreases, resulting in the accumulation of NICD, which phosphorylates ULK1 and then prevents the initiation of autophagy (Fig. 8). Based on this new finding, strategies designed to either prevent the EMT or activate autophagy may exert an effect on the other process, and targeting both processes may enhance their effectiveness.

## Data Availability

The data used and/or analyzed during the current study are available from the corresponding author on reasonable request.

## Abbreviations

EMT: Epithelial-mesenchymal transition
LECs: Lens epithelial cells
α-SMA: α-Smooth muscle actin
NICD: Notch intracellular domain
LC3: Microtubule-associated protein light chain 3
UBA: Ubiquitin-associated
LIR: LC3-interacting region
mTOR: Mammalian target of rapamycin
ULK1: Unc-51-like kinase 1
STZ: Streptozotocin
HE: Hematoxylin and eosin
DAPT: N-(N-[3,5-difluorophenacetyl]-1-alanyl)-S-phenylglycine t-butyl ester
TEM: Transmission electron microscope
